# Neural responses to acute stress predict chronic stress perception in daily life over 13 months

**DOI:** 10.1038/s41598-023-46631-w

**Published:** 2023-11-15

**Authors:** Marina Giglberger, Hannah L. Peter, Gina-Isabelle Henze, Elisabeth Kraus, Christoph Bärtl, Julian Konzok, Ludwig Kreuzpointner, Peter Kirsch, Brigitte M. Kudielka, Stefan Wüst

**Affiliations:** 1 Department of Psychology, University of Regensburg, Universitätsstraße 31, 93053 Regensburg, Germany; 2 Research Division of Mind and Brain, Department of Psychiatry and Psychotherapy CCM, Charité-Universitätsmedizin Berlin, Corporate Member of Freie Universität Berlin, Humboldt-Universität Zu Berlin, and Berlin Institute of Health, Berlin, Germany; 3 Department of Psychology, Computational Modeling in Psychology, Ludwig Maximilian University of Munich, Munich, Germany; 4 Department of Epidemiology and Preventive Medicine, University of Regensburg, Regensburg, Germany; 5 Department of Clinical Psychology, Central Institute of Mental Health, Medical Faculty Mannheim, University of Heidelberg, Heidelberg, Germany

**Keywords:** Psychology, Stress and resilience

## Abstract

The importance of amygdala, hippocampus, and medial prefrontal cortex (mPFC) for the integration of neural, endocrine, and affective stress processing was shown in healthy participants and patients with stress-related disorders. The present manuscript which reports on one study-arm of the LawSTRESS project, aimed at investigating the predictive value of acute stress responses in these regions for biopsychological consequences of chronic stress in daily life. The LawSTRESS project examined law students either in preparation for their first state examination (stress group [SG]) or in the mid-phase of their study program (control group [CG]) over 13 months. Ambulatory assessments comprising perceived stress measurements and the cortisol awakening response (CAR) were administered on six sampling points (t1 = − 1 year, t2 = − 3 months, t3 = − 1 week, t4 = exam, t5 =  + 1 week, t6 =  + 1 month). In a subsample of 124 participants (SG: 61; CG: 63), Scan*STRESS* was applied at baseline. In the SG but not in the CG, amygdala, hippocampus, and (*post-hoc* analyzed) right mPFC activation changes during Scan*STRESS* were significantly associated with the trajectory of perceived stress but not with the CAR. Consistent with our finding in the total LawSTRESS sample, a significant increase in perceived stress and a blunted CAR over time could be detected in the SG only. Our findings suggest that more pronounced activation decreases of amygdala, hippocampus, and mPFC in response to acute psychosocial stress at baseline were related to a more pronounced increase of stress in daily life over the following year.

## Introduction

While our knowledge on mechanisms mediating the link between chronic stress exposure and disease risk is still fragmentary, it appears undisputed that individual differences in the brain’s interpretation of and response to external and internal stressors constitute a significant determinant of vulnerability for or resilience to stress-related pathology^1^. Although animal models have substantially contributed to our understanding of stress processing in the central nervous system (CNS) and of the interplay with other systems including the hypothalamus–pituitary–adrenal (HPA) axis^2–4^, they cannot necessarily be transferred to humans^5,6^. Research on stress regulation in the human CNS has been facilitated by the advent of psychosocial stress paradigms, such as the Montreal Imaging Stress Task (MIST)^7^ or Scan*STRESS*^5,8^, reliably inducing both, robust neural and HPA axis responses in a functional magnetic resonance imaging (fMRI) environment. While some differences between animal and human models became evident^6^, an involvement of (pre)limbic regions, such as amygdala, hippocampus, and medial prefrontal cortex (mPFC), well-established in animals, could be confirmed in humans as well^2–5,9^. The importance of these brain regions for the integration of CNS and neuroendocrine stress processing is further highlighted by their repeatedly found relation with individual cortisol responses to acute stressors^5,9–11^. Moreover, subjective stress ratings were associated with neural activation changes in response to stress in amygdala, hippocampus, and different subregions of the prefrontal cortex as well^5,12–14^. In a conjunction analysis, the ventromedial PFC (vmPFC) was revealed as region of common activation for cortisol and subjective reactivity to acute stress, suggesting an integrative role of the vmPFC in endocrine and affect regulation^14^. Acute and chronic stress regulation in general and the integration of CNS, endocrine, and affective stress processing in particular, depends on limbic (most notably but not exclusively on amygdala and hippocampus) and prefrontal brain regions^3,5,9–15^. In patients with stress-related disorders including major depressive disorder, anxiety, or posttraumatic stress disorder (PTSD), functional and structural alterations in the same brain regions have been observed^16–21^. In addition, neural acute stress responses in various patient groups differed from those in healthy subjects with changes predominantly found in limbic and striatal-prefrontal regions^21–23^. For instance, in patients suffering from major depressive disorder, altered responses to acute psychosocial stressors like the MIST or Scan*STRESS* have been repeatedly observed in cortico-limbic regions including amygdala, hippocampus, vmPFC, and dorsolateral prefrontal cortex^22,23^. A history of childhood maltreatment was reported to augment the effects^24,25^. Although these cross-sectional findings are important, longitudinal designs would allow to contribute substantially to the investigation of mechanisms mediating the shift from acute to chronic stress and, eventually, to the evolvement of stress-related diseases. Demonstrating that differences in the brain's neural response to stress can be observed before the manifestation of signs of chronic stress could serve as one important prerequisite for the assumption of a causal link.

Therefore, the so-called brain-as-predictor approach appears promising in biopsychological stress research. It aims at revealing connections between neural activity in laboratory contexts and longer-term, ecologically valid outcomes^26^. To date, no study has used this approach to investigate the link between acute stress responses and chronic stress outcomes or health consequences. However, for other research questions, it has been already proven as valuable. For instance, Shapero et al.^27^ used this approach and discovered that resting state connectivity measures significantly predicted the onset of depression 3 to 4 years later. Another study by Urry et al.^28^ found activation patterns in the amygdala and prefrontal regions during an emotion regulation task to be related to diurnal cortisol slopes assessed later at home.

The current study focused on the investigation of the predictive value of acute neural stress responses on chronic stress outcomes in real life. It is part of the LawSTRESS project, a prospective-longitudinal study on long-lasting academic stress in a homogenous cohort. It examined German law students either in preparation for their first state examination (stress group [SG]) or without upcoming particular stress exposure (control group [CG]) over 13 months. Besides a suitable cohort, a valid multidimensional stress assessment with methods appropriate for usage in everyday life was essential for the implementation of such a longitudinal study. Thus, we used repeated ambulatory assessments (AA), allowing an ecologically valid recording of momentary stress-relevant experience and a higher reliability due to repeated real-time and real-life measurements^29^. The AA consisted of perceived stress ratings combined with the collection of saliva samples after awakening to measure the cortisol awakening response (CAR). The CAR represents a distinct increase of cortisol levels in the first 30 to 45 min after morning awakening^30,31^ and differs from the basal diurnal secretion pattern^32^. It is modulated by the suprachiasmatic nucleus (SCN) via the HPA axis and the sympathetic nervous system^33^. The CAR was found to be associated with a wide range of psychosocial and health-related variables, making it a promising tool in psychobiological stress research^31^. So far, it is unknown if neural stress responses are a significant predictor of CAR trajectories over a longer stress period in healthy participants. However, the CAR was indeed shown to be related to indicators of chronic stress. While in early reports findings have been mixed, a current review stated that studies with more reliable methodologies predominantly found associations between chronic stress and an attenuated CAR^34^. Chronic stress, in turn, was shown to be related to alterations in limbic and prefrontal regions^3,15^.

Based on this, the current study arm of the LawSTRESS project sought to test the hypothesis that responses to acute psychosocial stress in amygdala, hippocampus, and mPFC are significantly associated with changes in perceived stress and the CAR due to long-lasting exam stress. As the fMRI scan has been performed at baseline, a significant difference between the two groups (SG vs. CG) regarding neural and salivary cortisol responses have not been expected. Moreover, as suggested by the brain-as-predictor approach and as supplementary objective, we intended to contribute to the ecological validation of the used stress paradigm.

## Methods and materials

### Sample

Law students in preparation for their first state examination were assigned to the chronic stress group (SG) as it is considered as one of the longest and most stressful exam periods in the German university system. Students with a typical workload in the mid phase of their study program constituted the control group (CG). The present manuscript reports on data from a LawSTRESS subsample including all 124 (SG: 61; CG: 63) participants who underwent MRI (for a description of the entire study sample see Giglberger et al.^35^). Of these, six did not complete the entire study protocol. For all fMRI analyses, 13 participants were excluded due to pronounced motion artifacts after motion correction (i.e., absolute movement > 3 mm during at least one run; *n* = 12) or due to poor image acquisition (*n* = 1), resulting in 111 participants (fMRI sample). Demographic information is presented in Table 1.

**Table 1:** Demographic characteristics of the sample included in the analyses of the present report.

	Total sample		fMRI sample	
	Stress group	Control group	Stress group	Control group
*n*	61	63	56	55
Age (mean ± standard deviation)	22.73 (± 1.58)	21.10 (± 2.05)	22.75 (± 1.61)	21.16 (± 2.05)
Women	*n* = 36 (59.0%)	*n* = 45 (71.4%)	*n* = 33 (58.9%)	*n* = 37 (67.3%)

Women not using hormonal contraceptives (19.8%; no statistically significant difference between groups, *p* = 0.533) were scheduled for the MRI session during the luteal phase of the menstrual cycle^36^, confirmed by urinary ovulation test kits (gabmed GmbH, Köln, Germany).

Individuals who met any of the following (self-reported) criteria were not included: current psychiatric, neurological, or endocrine disorders, treatment with psychotropic medications or any other medication affecting CNS or endocrine functions, regular night-shift work or MRI-scanner contraindications. The study was conducted in accordance with the Declaration of Helsinki and approved by the ethics committee at the University of Regensburg, Germany (“Ethikkommission der Universität Regensburg”; https://www.uni-regensburg.de/ethikkommission/startseite/index.html). All participants provided written informed consent and received monetary compensation as well as individualized feedback on their study results.

### General procedure

A detailed overall description of the LawSTRESS project can be found elsewhere (https://epub.uni-regensburg.de/51920/^35^). For the present report, the following aspects are relevant: The protocol comprised six sampling points (t1–t6) over 13 months. T1 for the SG was 1 year prior exam. The remaining appointments were 3 months (t2) and 1 week (t3) prior exam, at the middle of the 8-days exam period (t4), as well as 1 week (t5) and 1 month (t6) thereafter. The same procedure, except t4 (exam), applied to the CG. Data collection was carried out in different cohorts and lasted from March 2018 until April 2021.

**Figure 1:**
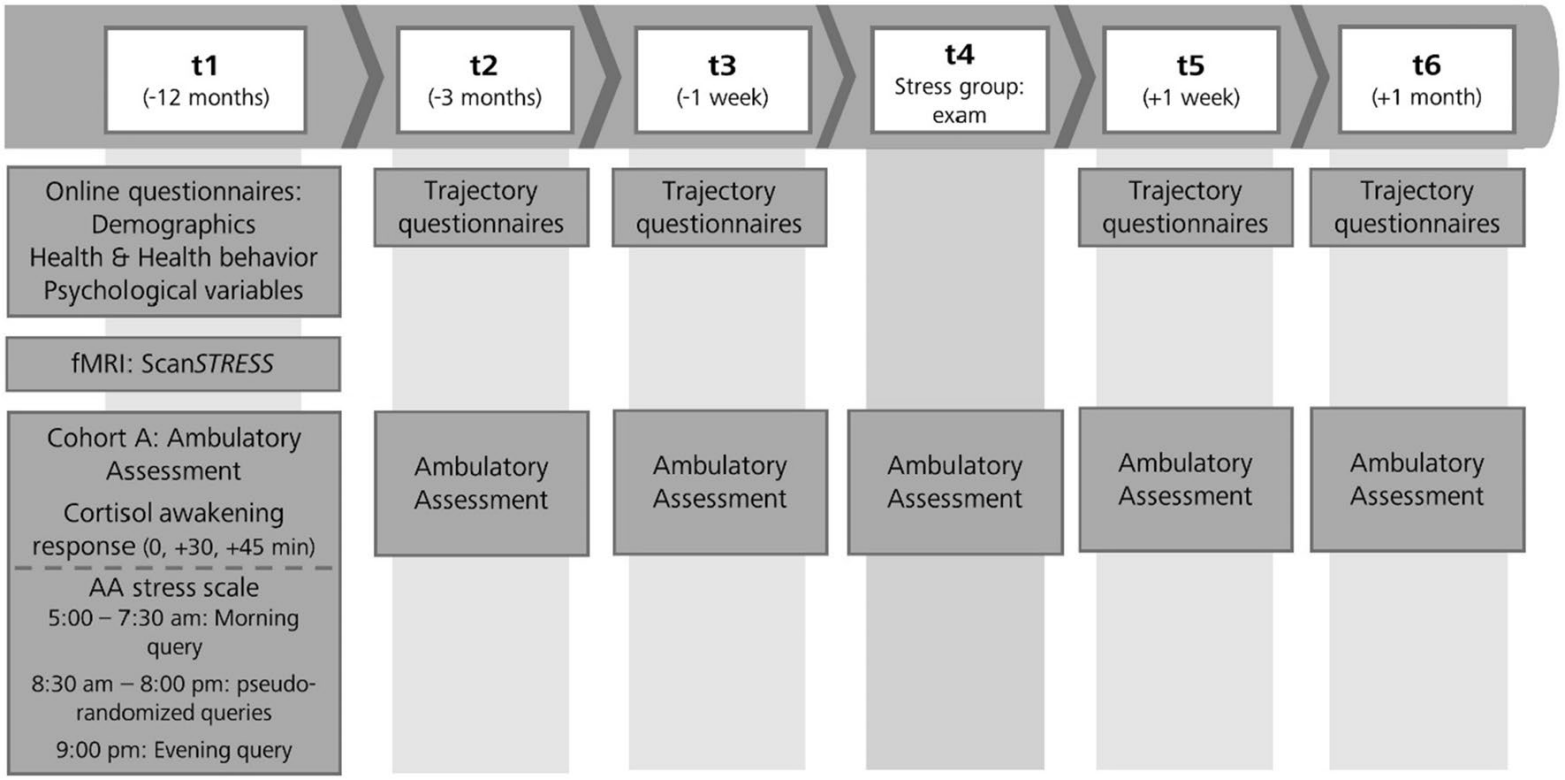
Timing of the data collection for the ambulatory assessment and the MRI measurement. For an overview of the entire study procedure of the LawSTRESS project see https://epub.uni-regensburg.de/51920/.

At t1, an online questionnaire battery was submitted via SoSci Survey (https://www.soscisurvey.de/^37^) to assess baseline data, psychometrics, physical health, health behavior, and university studies-related variables. Subsequently, an appointment at the laboratory was arranged to hand out the material for the first AA (including assessment of the CAR) along with a detailed instruction. During a second appointment at the laboratory, an MRI scanning session (including the stress paradigm) took place for the MRI subsample. Further AA were conducted at t2 to t6. In the present manuscript, only AA (including the CAR) and fMRI data are presented (Fig. 1).

### Ambulatory assessment

A detailed description of the AA can be found in Supplementary Methods section “Ambulatory assessment (AA)”. In brief, the AA consisted of an assessment of momentary perceived stress with the five-items AA stress scale^35^ ten times a day (movisensXS; version 1.3.2 to 1.5.13; movisens, Karlsruhe, Germany) and the assessment of the CAR. At t1, t2, t5, and t6, the AA was conducted on two consecutive working days, while at the sampling points close to or during the examination days (t3 and t4), it took place on single days only. On the first day of each AA, participants collected three saliva samples (Salivettes^®^, Sarstedt, Nuembrecht, Germany) for later CAR assessment immediately after awakening and 30 and 45 min later. Only at t1, we assessed the CAR on both sampling days.

### Scan*STRESS*

Scan*STRESS* is a stress induction paradigm for fMRI conditions developed by our group, predominantly aiming at inducing social-evaluative threat and uncontrollability as stress-inducing psychological components^5,8^. Briefly, this block design paradigm consists of two runs containing two conditions (stress vs. control) each. During stress blocks, participants are prompted to perform visually presented arithmetic and mental rotation tasks under time pressure. Task speed and difficulty are adapted to the participant’s performance ensuring frequent failure and uncontrollability. After trials and between runs, a previously introduced observation panel gives standardized negative feedback regarding work speed and error frequency. During control blocks, simple figure and number matching tasks have to be performed in the absence of time pressure, observation and negative feedback. Saliva samples were collected at ten timepoints (t = − 75, − 15, − 1, + 15, + 30, + 50, + 65, + 80, + 95, + 110 min relative to stress onset). Test sessions were scheduled between 1:00 and 5:00 p.m. For a detailed overview of the used Scan*STRESS* protocol see Supplementary Methods section “Scan*STRESS* protocol” and Henze et al.^5^.

### fMRI data acquisition and analysis

Imaging data was acquired on a Siemens MAGNETOM Prisma 3T MRI scanner (Siemens Healthcare, Erlangen, Germany) equipped with a 64-channel head coil. Functional images were acquired using a series of blood-oxygenation-level-dependent gradient echo planar imaging images (EPI) with the following parameters: TR = 2000 ms, TE = 30 ms, flip angle = 90°, matrix size = 64 × 64 mm^2^, FoV = 192 mm, 37 slices, slice thickness = 3 mm, 1 mm gap, voxel size = 3 mm isotropic, interleaved. The following parameters were used for anatomical pictures: TR = 2400 ms, TE = 2.18 ms, flip angle = 9°, voxel size = 0.8 mm isotropic, distance factor = 50%. The complete MRI session included two resting state sequences after the stress paradigm which are beyond the scope of this manuscript.

Data analyses were carried out with FSL 6.0^38,39^ using FEAT version 6.0^40,41^. The first five EPI volumes were discarded to account for inhomogeneities of the magnetic field. For a detailed description of the pre-statistics processing and the used analysis steps see Henze^42^. *Z* (Gaussianized *t/F*) statistic images were thresholded a priori non-parametrically using clusters determined by z > 2.3.

For each participant, general linear models (GLMs) were defined containing a total of 12 regressors—six conditions (stress arithmetic subtraction, stress figure rotation, control numbers, control figures, announcement of stress, announcement of control) and six motion regressors. In a first step, one GLM was computed for each participant and each run (first level analysis, z > 2.3) to account for scanner drifting. Next, we analyzed mean responses for each participant over both runs (second level, z > 2.3). On a third level, a group analysis (mixed effects, z > 2.3) was conducted to study the overall neural stress response^5^. For the main task effects (stress > control; control > stress), corrections via the familywise error (FWE) for multiple comparison at a significance level of *p* < 0.025 (two-tailed combined test FWE *p* < 0.05) were applied.

**Figure 2:**
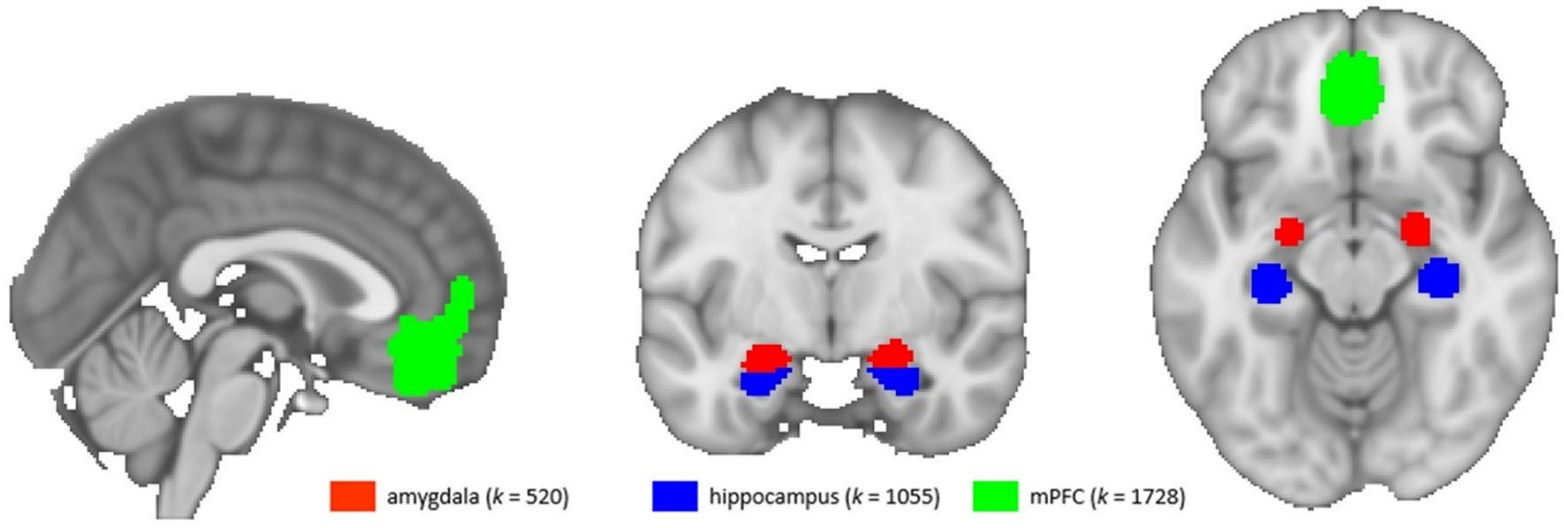
Masks of the used regions of interest amygdala, hippocampus, and medial prefrontal cortex (mPFC). *k* = number of voxels.

To test the predictive value of certain brain regions on the trajectory of our stress measures, we extracted mean beta values of our a priori defined regions of interest (ROIs) amygdala, hippocampus, and medial prefrontal cortex (mPFC) from second level analysis (stress > control). For amygdala and hippocampus, we used masks from the Harvard–Oxford Atlas; for mPFC, we created a mask from the deactivation pattern found in Henze et al.^5^ associated with the anatomical mPFC region (Fig. 2). ROI-analyses were performed using fslmaths and featquery.

### Statistical analyses

Data were analyzed using R (version 4.0.3, R Core Team, 2020), the rstatix^43^, glmmTMB^44^, nlme^45^ and MuMIn^46^ packages. All models were estimated with Maximum Likelihood and the significance level was set at α = 0.05.

#### Scan*STRESS* cortisol response

For the acute stress response, log-transformed cortisol values served as within-subject factors and sex as between-subject factor in a repeated measures ANOVA. Greenhouse–Geisser corrections were applied where appropriate and only adjusted results are reported.

#### AA stress scale and the CAR

The time course of the AA stress scale was calculated using generalized linear models. In this two-level model, the variable *group* (CG = 0; SG = 1), the variable *timepoint* as linear, quadratic, and cubic trend and the interactions between these time trends and *group* were included allowing a different curvilinear time course for each group. AA values were clustered in participants, hence random intercepts and slopes for timepoint by participant were estimated to account for dependencies in the data.

The model testing the predictive value of our ROIs on the rise of perceived stress until the exam comprised the timepoints t1–t4. The continuous variable *ROI* (*amygdala*, *hippocampus,* and *mPFC*) was included in separate models (due to different time trends; see 3.2) for SG and CG as main effect and in interaction with the time trends (SG: *timepoint*, *timepoint*^*2*^; CG: *timepoint*). For explorative analyses, models for the unilateral ROIs were calculated additionally.

To test for alterations of the CAR in the SG, we computed three level linear mixed models with cortisol measurements (level 1) nested within timepoints (level 2), nested within participants (level 3). We added random intercepts for both, participants and timepoints, as well as random slopes for minutes (0, 30, and 45 min after awakening). The final model contained the variables *timepoint* (t1–t6) and *minutes* as well as the covariates *hormonal status* (dummy-coded; reference category: women not using hormonal contraceptives) and *awakening time* in minutes (person-mean centered). Cortisol data was log-transformed.

The analyses testing the predictive value of our ROIs on alterations of the CAR comprised the timepoints t1–t4. The ROIs were added separately as main effects, in interaction with *minutes* and *timepoint* and as three-way interaction (*minutes *×* timepoint *×* ROI*) in separate models for SG and CG.

## Results

### Scan*STRESS*: cortisol and neural responses

**Figure 3:**
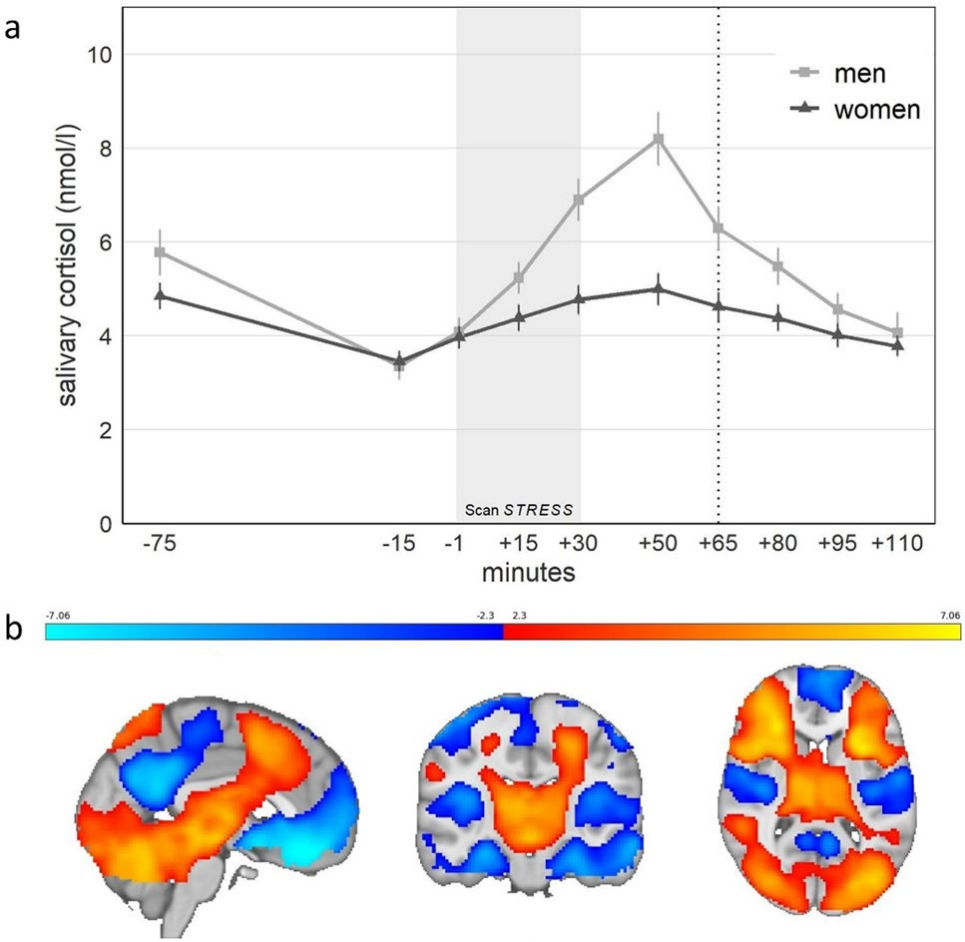
Responses to Scan*STRESS*. (**a**) Salivary cortisol responses in women and men (± SEM) (dotted line = end of the scanner session). (**b**) Activations (red to yellow) and deactivations (blue) in response to psychosocial stress induction (z > 2.3).

On average, cortisol levels showed a significant rise in response to acute laboratory stress exposure (*F*_3.29, 388.19_ = 32.69, *p* < 0.001, η^2^ = 0.22). As expected, men showed higher mean responses than women (*F*_3.29, 388.19_ = 9.22, *p* < 0.001, η^2^ = 0.07). However, the female subsample also exhibited a significant cortisol rise (*F*_3.07, 239.52_ = 7.46, *p* < 0.001, η^2^ = 0.09; Fig. 3a). During Scan*STRESS*, activations (stress > control) emerged in a cluster including the bilateral insula, frontal, and occipital regions, whereas deactivations (control > stress) arose in two clusters comprising, among others, medial regions of the PFC, hippocampus, and amygdala (all in cluster1) and angular gyrus (cluster2; two-tailed combined FWE-corrected *p* < 0.05; Fig. 3b; for peak voxels see Supplementary Table S1). Mean beta values of the a priori defined ROIs amygdala, hippocampus, and mPFC (all bi- and unilateral) are shown in Table 2. As expected, no significant differences were found between SG and CG, neither for the cortisol nor the fMRI analyses.

**Table 2:** Mean beta values ± *SD* of the main task effect stress > control for the a priori defined ROIs amygdala, hippocampus, and mPFC (bi- and unilateral).

		*n*	Mean	*SD*
Amygdala	Bilateral	109 (SG: 56)	− 0.09	0.30
	Left	108 (SG: 56)	− 0.09	0.31
	Right	109 (SG: 55)	− 0.07	0.30
Hippocampus	Bilateral	111 (SG: 56)	− 0.11	0.26
	Left	111 (SG: 56)	− 0.12	0.29
	Right	111 (SG: 56)	− 0.10	0.24
mPFC	Bilateral	85 (SG: 42)	− 0.22	0.48
	Left	88 (SG: 44)	− 0.24	0.49
	Right	80 (SG: 40)	− 0.19	0.48

*SD* = standard deviation.

### Association between neural responses and perceived stress

On average, participants who completed at least the first timepoint responded to 89.01 out of 100 AA stress scale queries. A model containing a cubic trajectory represented the best model fit (compared to the preceding model: linear model ∆AIC = 2227.52; quadratic model ∆AIC = 697.02; cubic model ∆AIC = 142.53). The trajectory of perceived stress levels differed significantly between groups (*timepoint × SG b* = 0.39, *p* < 0.001; *timepoint*^*2*^* × SG b* = − 0.20, *p* < 0.001; *timepoint*^*3*^* × SG b* = 0.02, *p* < 0.001). In the SG, mean perceived stress increased until the exam and showed a decline thereafter. Stress levels in the CG stayed relatively stable (Supplementary Fig. S1 and Supplementary Table S2). There was no significant difference between groups at t1 (*SG b* = 0.10, *p* = 0.074).

Testing our hypotheses, only the timepoints until the exam (t1–t4) especially in the SG (SG.model) were of interest. Nevertheless, also models for the CG (CG.model) were calculated to explore potential influences of the ROIs on perceived stress levels without particular stress exposure. A quadratic trajectory for the SG.model (compared to the preceding model: linear SG.model ∆AIC = 740.38; quadratic SG.model ∆AIC = 174.44) and a linear one for the CG.model (compared to the preceding model: ∆AIC = 421.01; *timepoint b* = 0.01, *p* = 0.413) represented the best fit.

#### Amygdala

**Figure 4:**
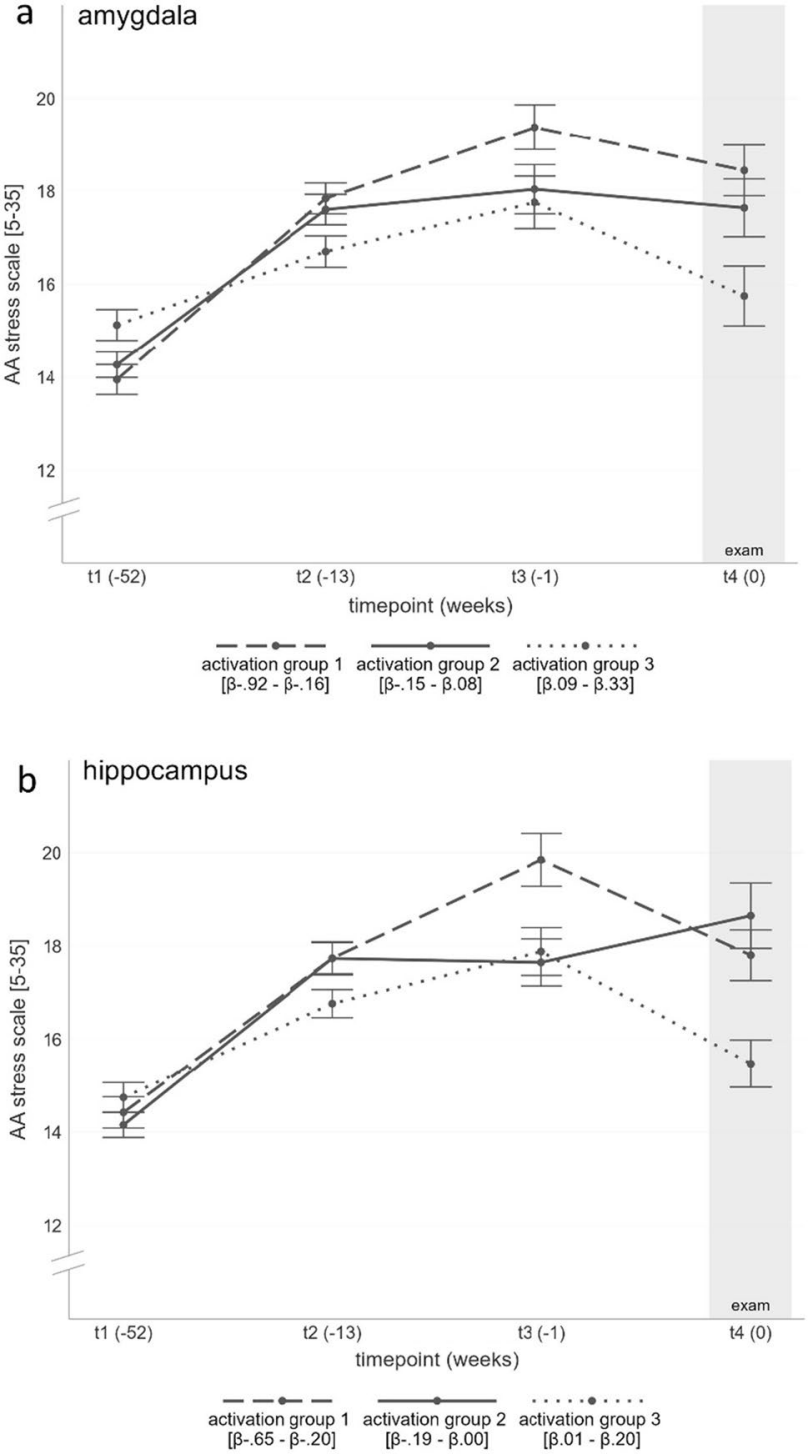
Time course of the *AA stress scale* (± SEM) in the stress group. For illustrative purposes, participants were divided into three groups of equal size according to their amygdala (**a**) and hippocampus (**b**) responses (indicated by β values), respectively. t = timepoint; t1 = 1 year before the exam, t2 = 3 months prior exam, t3 = 1 week prior exam and t4 = in the middle of the exam period.

Entering the beta values of the amygdala activation to the SG.model led to an improvement (ΔAIC = 4.53). They significantly predicted the increase of stress perception until the exam. In detail, a stronger decrease in amygdala activation to acute stress was related to a steeper curve of perceived stress levels and a peak closer to t4 (Fig. 4a and Table 3). In an explorative analysis, this result was found in both hemispheres (timepoint × left amygdala *b* = − 0.21, *p* = 0.006; timepoint^2^ × left amygdala *b* = 0.06, *p* < 0.001; timepoint × right amygdala *b* = − 0.21, *p* = 0.017; timepoint^2^ × right amygdala *b* = 0.05, *p* = 0.004). Entering beta values of the amygdala response to the CG.model did not lead to an improvement (ΔAIC = − 0.95).

**Table 3:** Parameter estimates for overall effects for the final SG.model with amygdala or hippocampus activation as predictor.

Fixed effects	Estimate		*SE*		Significance	
	Amygdala	Hippocampus	Amygdala	Hippocampus	Amygdala	Hippocampus
Intercept	2.64	2.63	0.04	0.04	** < 0.001**	** < 0.001**
Timepoint	0.22	0.21	0.02	0.03	** < 0.001**	** < 0.001**
Timepoint^2^	− 0.06	− 0.06	0.01	0.01	** < 0.001**	** < 0.001**
ROI	0.07	− 0.01	0.13	0.16	0.578	0.956
Timepoint × ROI	− 0.21	− 0.22	0.08	0.10	**0.009**	**0.025**
Timepoint^2^ × ROI	0.05	0.06	0.02	0.02	**0.002**	**0.006**

Random effects	*SD*		Correlation intercept			
	Amygdala	Hippocampus	Amygdala	Hippocampus		
Participant (Intercept)	0.28	0.28				
Timepoint	0.13	0.13	− 0.27	− 0.28		

*ROI =* region of interest, *SE* = standard error, *SD* = standard deviation.

Significant values are in bold.

#### Hippocampus

Adding the beta values of the hippocampus activation also improved the SG.model (ΔAIC = 2.52) with similar results as for the amygdala (Fig. 4b and Table 3). Explorative analyses indicated a predictive value for the right hippocampus (timepoint x right hippocampus *b* = − 0.28, *p* = 0.008; timepoint^2^ × right hippocampus *b* = 0.07, *p* = 0.002), but not for the left (no model improvement ΔAIC = − 0.41). The CG.model showed no improvement after entering the hippocampus beta values (ΔAICs = − 1.14).

#### mPFC

Since neither the SG.model nor CG.model parameters improved after adding the beta values of the mPFC (SG.model: ΔAIC = − 3.23; CG.model: ΔAIC = − 0.50), no significant influence of this ROI was assumed. However, in an explorative analysis, an effect of the right mPFC on the trajectory of the SG could be found (timepoint^2^ × right mPFC *b* = 0.02, *p* = 0.008; Supplementary Fig. S2).

### Association between neural responses and the CAR

The models for the CAR analysis within the SG comprised 61 students of whom 55 completed the whole study protocol. Consistent with our finding in the complete SG sample^35^, a blunted CAR was found at t4 (*t4* × *30 min b* = − 0.12,* p* = 0.002; *t4* × *45 min b* = − 0.18, *p* < 0.001; Supplementary Fig. S3 and Supplementary Table S3). Adding beta values of the ROI activations as predictors led neither in the models for the SG (amygdala: ∆AIC = − 8.34; hippocampus: ∆AIC = − 10.68; mPFC: ∆AIC = − 22.08) nor in the ones for the CG (amygdala: ∆AIC = − 17.90; hippocampus: ∆AIC = − 19.44; mPFC: ∆AIC = − 17.16) to improvements. Thus, no significant associations between activation patterns in our ROIs and alterations of the CAR could be detected.

## Discussion

For stress research, animal models are indisputably relevant, but their findings cannot necessarily be directly transferred to humans. Stress paradigms like restraint stress, commonly used in animals, show substantial differences compared to human stress experiences in laboratories and everyday life regarding stress intensity, duration, and stress inducing psychological components. Nevertheless, the importance of amygdala, hippocampus, and mPFC for the integration of CNS, endocrine, and affective stress processing, first documented in rodents^2–4,47^, was confirmed in studies with healthy participants^5,9,11,13^ and patients with major depression, anxiety, or PTSD^16–20^. The overarching aim of the present study was to explore if stress processing in these regions is related to later stress responses outside the laboratory. Therefore, we assessed the predictive value of neural responses to acute psychosocial stress in amygdala, hippocampus, and mPFC for biopsychological consequences of chronic stress exposure in daily life. Due to their relevance for a meaningful investigation of this research question, two features of our study design should be emphasized^26^. First, we used a protocol that reliably induces psychosocial stress in the MRI scanner^5,8^. The observed cortisol responses support the assumption that neural activation changes were indeed responses to psychosocial stress and not mere indicators of task-related mental load. Our ROIs amygdala, hippocampus, and mPFC show on average decreased activation, hence activation was higher during control than stress conditions. This is in line with previous findings examining neural responses to acute stress and, in particular, their relation to cortisol responses^5,9,48^. Limbic regions play an important role in the regulation of the HPA axis stress response. It is assumed that their active state during rest leads to a tonic inhibition of HPA axis activity, changing to a disinhibition facilitating stress hormone release after stress onset^9,48^. Ongoing processing and contextualization of potentially threatening sensory information might be a default mode of limbic regions which might be disrupted as a response to a stimulus perceived as threatening, thus promoting the focus on the threatening task at hand^9,49^.

The second key feature is the longitudinal and detailed assessment of stress-related variables in everyday life allowing prospective registration of changes over time. In the stress group, we found significant increases in stress levels in everyday life until the exam, whereas non-exam students stayed relatively stable. The perceived stress trajectory of the SG could be predicted by individual activation changes in amygdala and hippocampus; the higher the decrease of activation in these ROIs during acute stress at baseline, the greater the perceived stress increase until the exam 12 months later. Consistent with our longitudinal data, lower hippocampus activation during the MIST was recently reported to be related to scores on a daily life stress scale in a cross-sectional study^50^. Associations with the mPFC could not be observed in our study which might partly be due to a decreased number of participants in this model. However, an explorative analysis suggested that the right mPFC was putatively related to the perceived stress changes in the SG. Remarkably, the unilateral analyses for the hippocampus also revealed only a significant association with the right hemisphere which was repeatedly found to be associated with negative emotions^51–54^. Additionally, stress seems to stimulate greater right hemispheric involvement^55^. Amygdala activation and hippocampus volume were shown to predict depression and PTSD symptoms^56–58^. Complementing these clinical findings, the present study suggests that also in the healthy brain, limbic responses to stress-related content may serve as predictor for the experience of chronic stress.

Moreover, with all due caution, we would like to offer the speculation that our study provided evidence for a certain individual stability of stress processing over time and contexts: Over time, as acute neural stress responses were associated with perceived stress reactions 1 year later; and over contexts, since the experience of acute stress in a scanner differs from the experience of daily hassles and ongoing stressors in real life. The idea that stress processes are to some extent stable, is supported by genetic studies reporting significant heritabilities for volumes of subcortical regions, like amygdala and hippocampus (e.g.,^59^). Also, their reactivity to negative stimuli and functional coupling with other stress-relevant brain regions seem to be under genetic influence^60–62^.

No associations between activation changes in our ROIs and stress levels at baseline or the trajectory of the perceived stress levels in the CG could be detected. This finding supports the view that interindividual differences in stress processing mainly become visible when stress response systems are challenged. Consistent with this notion, internalizing symptoms 1 to 4 years after an fMRI session could be predicted by threat-related amygdala reactivity, but only in participants experiencing relatively high life stress during this time period^63^.

Furthermore, we asked if our data contribute to research on the ecological validity of laboratory stress paradigms. From that perspective, the present findings are indeed encouraging. They provide novel evidence for a significant association between acute responses to psychosocial challenge in brain regions involved in stress processing and a longer-term, ecologically valid outcome, namely the development of chronic stress symptoms in real life.

Regarding cortisol awakening responses, SG participants showed, compared to their baseline, a significant decrease during the examination days (t4). We assume that the blunted CAR at t4 can be interpreted as a temporary hypocortisolism in otherwise healthy, young students^35^. An association between neural stress response patterns and alterations in the CAR could not be detected. Convincing evidence for an influence of amygdala, hippocampus, and mPFC on HPA axis stress responses has been found in animal as well as human studies^2–4^. Moreover, while the regulation of the CAR was shown to be modulated by prelimbic regions^33,64^ and associations between the CAR and chronic stress have been found^34^, it was mainly reported to be unrelated to cortisol reactivity to experimentally-induced psychological stress^65^. This is in line with the assumption that the CAR is modulated by additional regulatory mechanisms, for instance a direct influence of the adrenal cortex by the suprachiasmatic nucleus^33^. This and perhaps other mechanisms may partly explain why on the one hand academic stress in our study affected the CAR, and why on the other hand, this alteration was not associated with activation changes in our limbic ROIs.

Our study has some limitations that need to be considered. First, we cannot rule out a certain selection bias in our sample. Students who already felt stressed by their regular study program and who anticipated an exceedingly stressful exam phase did possibly not volunteer to participate in a study that was related to (modest) additional burden. Therefore, it might be possible that we underestimated the mean stress load in the SG to a certain extent. Second, although we applied several methods to increase the quality of our CAR assessment (electronic monitoring devices, random codes, encouragement to report non-compliance), a reliable technique to verify the exact awakening time was not available in the present study. Thus, a confounding effect to a certain extent cannot be ruled out. However, at least a group-specific effect of this potential confounder appears unlikely as a delay between awakening and collecting the first sample should result in erroneously high cortisol levels at awakening. This was not observed in our study^35^. Third, missing data in our ROI analyses led to different sample sizes in the models including amygdala (*n*_SG_ = 56; *n*_CG_ = 53), hippocampus (*n*_SG_ = 56; *n*_CG_ = 55), and mPFC (*n*_SG_ = 42; *n*_CG_ = 43). Finally, while the detailed protocol and information on previous publications of the LawSTRESS project have been made easily accessible (https://epub.uni-regensburg.de/51920/), the study was not preregistered.

Taken together, the LawSTRESS project was designed as prospective-longitudinal study and used a multimethod and multidimensional approach to assess chronic academic stress. While the experience of this first state examination is specific for German law students, it can be assumed that the overall psychosocial profile of this stressful period is generally comparable to stress that individuals frequently experience in schools, universities, or at the workplace. We found that a more pronounced decrease of activation in amygdala and hippocampus in response to acute psychosocial stress was related to a more pronounced increase of stress over the following year. These neural responses could be indicators of an individual stress response pattern showing a certain stability over time and contexts and significantly predicting chronic stress perception in real life. In that sense, it appears promising to further pursue the idea in future studies that the described neural stress responses may serve as stress vulnerability or resilience marker. Our study supports the view that the brain-as-predictor approach can be a useful strategy in human psychobiological stress research.

### Supplementary Information

Supplementary Information.

## Data Availability

Datasets generated or analyzed during the study are available from the corresponding author on request.
