# Supplementary material for: Neural responses to acute stress predict chronic stress perception in daily life over 13 months
Source: Sci Rep. 2023 Nov 15;13:19990. doi: 10.1038/s41598-023-46631-w (PMC10651906; doi:10.1038/s41598-023-46631-w)
Supplement: Supplementary file 1 — Supplementary Information. [file 41598_2023_46631_MOESM1_ESM.pdf]

SUPPLEMENTS: Neural responses to acute stress predict chronic stress perception in daily life over 13 months

Marina Giglberger[1], Hannah L. Peter[1], Gina-Isabelle Henze[1,2], Elisabeth Kraus[3], Christoph Bärtl[1], Julian Konzok[1,4], Ludwig Kreuzpointner[1], Peter Kirsch[5], Brigitte M. Kudielka[1], Stefan Wüst[1]*

[1] Department of Psychology, University of Regensburg, Regensburg, Germany

[2] Research Division of Mind and Brain, Department of Psychiatry and Psychotherapy CCM, Charité-Universitätsmedizin Berlin, Corporate Member of Freie Universität Berlin, Humboldt-Universität zu Berlin, and Berlin Institute of Health, Berlin, Germany

[3] Department of Psychology, Computational Modeling in Psychology, Ludwig Maximilian University of Munich, Munich, Germany

[4] Department of Epidemiology and Preventive Medicine, University of Regensburg, Germany

[5] Department of Clinical Psychology, Central Institute of Mental Health, Medical Faculty Mannheim, University of Heidelberg, Heidelberg, Germany

* Correspondence concerning this article should be addressed to:

Stefan Wüst, Department of Psychology, Universitätsstraße 31, 93053 Regensburg, Germany.

Phone: +49 (0)941 943 5646, E-mail: stefan.wuest@ur.de

1. Supplementary methods 2
   1.1. Ambulatory assessment (AA) 2
   1.2. Scan*STRESS* protocol 3
2. Supplementary results 4
   2.1. Scan*STRESS*: neural responses 4
   2.2. Perceived stress 5
   2.3. Cortisol awakening response 7
References 9

1. Supplementary methods

1.1. Ambulatory assessment (AA)

The AA was carried out with the combined smartphone app and web platform movisensXS (version 1.3.2 to 1.5.13; movisens, Karlsruhe, Germany). For the MRI sample, it comprised an assessment of perceived stress levels ten times a day, a short morning and evening questionnaire, and the collection of three saliva samples after awakening for later assessment of the CAR.

To measure momentary perceived stress, 18 items covering positive/negative mood (e.g. “I am happy”), calm/restlessness (e.g. “I am tense”), concern/confidence (e.g. “I am worried”), self-satisfaction/shame (e.g. “I am disappointed”), overload (e.g. “I am under time pressure”), anxiety (e.g. “I am afraid”), and somatic symptoms (e.g. “I am in pain”) were assessed on a seven-point Likert scale. Item wording was predominantly based on existing questionnaires (Positive and Negative Affect Schedule [1]; Multidimensional Mood State Questionnaire [2]; State-Trait Anxiety Inventory [3]), or research papers [4]. Exploratory factor analyses of 10.0% of the surveys of the entire LawSTRESS sample (1613 out of 16430) revealed a one-factor-solution with the five items “time pressure”, “relaxed”, “tense”, “overstrained”, and “disappointed with the own performance”. Subsequent confirmatory factor analyses of all surveys showed good fit indices (all CFI > .99; all RMSEA < .15). Thus, for all analyses the five-items AA stress scale was used.

At t1, t2, t5, and t6, the AA was conducted on two consecutive working days while at the sampling points close to or during the examination days (t3 and t4), it took place on single days only. T4 in the SG (not in the CG) was carried out at the weekend between the eight-days exam period. The first daily query took place immediately at the individually chosen awakening time between 5:00 and 7:30 a.m. and the last one at 9:00 p.m. The remaining queries were presented at pseudo-randomized times between 8:30 a.m. and 8:00 p.m. with a minimum interval of 60 minutes between two queries. Across all measurement points, we collected 100 queries per participant. Those who did not have a compatible Android smartphone were equipped with a device provided by the institute.

The CAR was assessed on both sampling days at t1 and on the first day of each AA phase at the remaining timepoints. Saliva samples were collected immediately after waking as well as 30 and 45 minutes later using Cortisol Salivettes® (Sarstedt, Nuembrecht, Germany). Participants were instructed not to drink (except from water), eat, smoke, or brush teeth during the first 45 minutes after awakening. To increase compliance and sampling accuracy, functional and non-functional MEMS caps from the company AARDEX Ltd. (Zug, Switzerland) were used in 61.9% – 77.2% (varying over sampling points) of the measurements [5,6]. In addition, a random three-digit code was presented with the

reminder for saliva sampling via smartphone and participants were instructed to transfer the digits to the sampling tube.

Saliva samples were stored at −20°C until analysis and analyzed in duplicate using a time-resolved fluorescence immunoassay with fluorometric end-point detection (DELFIA) at the biochemical laboratory of the University of Trier [7]. The intra-assay coefficient of variation was between 4.0% and 6.7%; inter-assay coefficients of variation were between 7.1% and 9.0%.

1.2. Scan*STRESS* protocol

Scan*STRESS* is a stress induction paradigm for fMRI conditions developed by our group, predominantly aiming at inducing social-evaluative threat and uncontrollability as stress-inducing psychological components [8,9].

Participants arrived in the laboratory 75 minutes prior stress onset. After receiving instructions, a relaxation phase was implemented during which a neutral movie was presented. Forty-five minutes prior stress onset, a sugary drink (75 g glucose in 200 ml herbal tea) was given and approximately 20 minutes later a brief training session of the Scan*STRESS* control blocks was completed.

The paradigm was implemented in Presentation® software (version 12.9, www.neurobs.com) and consisted of two different conditions: a stress and a control condition. During stress blocks, participants had to solve visually presented arithmetic and spatial mental rotation tasks under time pressure. Task speed and difficulty were adapted to the participant's performance ensuring frequent failure and uncontrollability. Furthermore, a previously introduced observation panel gave feedback regarding working speed ("work faster") and accuracy ("error") via life stream. During the control condition, simple figure and number matching tasks had to be performed in the absence of time pressure and negative feedback (indicated by a black cross over the video stream).

Scan*STRESS* comprised two runs with a duration of 11:20 minutes each. The first one started with the control condition, the second one with the stress condition. In each run, stress and control blocks were presented in an alternating order. Each task block lasted 60 seconds and was preceded by a five seconds announcement phase and followed by a 23 seconds break. During the break between the two runs, the MRI scanner was paused and subjects received additional feedback that the committee was unsatisfied with their performance and that they needed to increase their efforts. Furthermore, biological (salivary cortisol, heart rate) and psychological (emotional stress reactivity) responses were repeatedly collected. The complete MRI session included two resting state sequences after the stress paradigm and an anatomical scan, which are beyond the scope of this manuscript. After completion of the MRI scan, participants remained in the laboratory for another 45 minutes to fill out questionnaires. Test sessions were scheduled between 1:00 and 5:00 p.m.

2. Supplementary results

2.1. Scan*STRESS*: neural responses

Table S1. Activated and deactivated structures under psychosocial stress (two tailed combined FWE-corrected for whole brain, threshold < .05) including z- and p-values as well as the localization of peak voxels.

**Brain structure**		**statistics**			**MNI coordinates**		
		k	***p***	**z**	**X**	**Y**	**Z**
**Insula**	**left**	**100415**	**< .001**	**6.92**	**-32**	**16**	**6**
	right			6.83	30	30	8
Superior frontal gyrus	right			6.81	22	-2	50
Middle frontal gyrus	right			6.80	30	4	48
Lateral occipital cortex	left			6.76	-18	-72	40
Middle frontal gyrus	left			6.73	-30	2	54
**Medial frontal cortex**	**right**	**31342**	**< .001**	**-7.26**	**2**	**40**	**-20**
	left			-6.92	-6	34	-26
Subcallosal cortex	left			-6.60	-2	10	-14
Inferior frontal gyrus, orbital	right			-6.42	20	26	-18
Precuneus	right			-6.32	0	-54	24
	left			-6.08	0	-66	28
**Angular gyrus**	**left**	**2361**	**.008**	**-6.79**	**-56**	**-66**	**34**
	left			-6.65	-54	-64	42
	left			-6.59	-54	-72	34
Inferior parietal gyrus	left			-5.30	-40	-80	46

Note. *k* = cluster size in voxels; MNI = Montreal Neurological Institute; global cluster maxima are in boldface.

2.2. Perceived stress

Table S2. Parameter estimates for overall effects for the final perceived stress model.

**Fixed Effects**	**Estimate**	***SE***	***p***
Intercept	2.51	0.04	**< .001**
Timepoint	0.03	0.02	.077
Timepoint^2	0.01	0.01	.522
Timepoint^3	-0.00	0.00	**.030**
SG	0.10	0.06	.074
Timepoint x SG	0.39	0.03	**< .001**
Timepoint^2 x SG	-0.20	0.01	**< .001**
Timepoint^3 x SG	0.02	0.00	**< .001**
**Random Effects**	*SD*	Correlation (Intercept)	
Subject (Intercept)	0.31		
Timepoint	0.06	-.20	

Note. SE = Standard error; *SD* = Standard deviation; SG = Stress group.

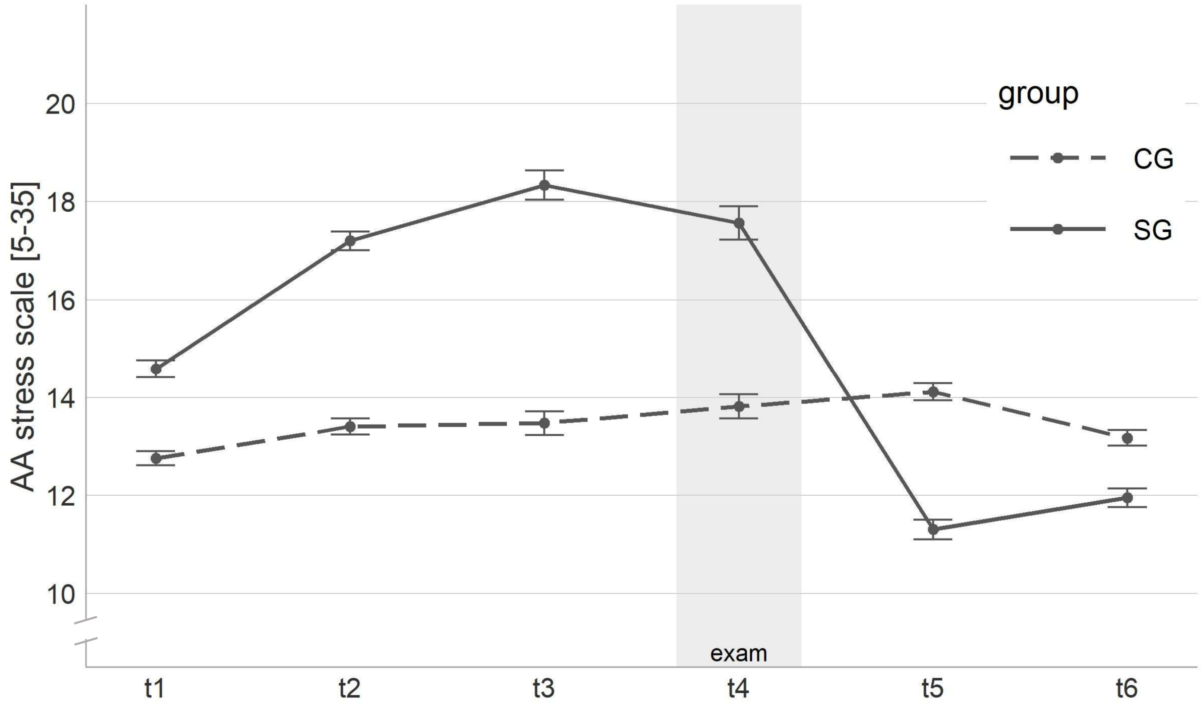

Figure S1. Time course of mean perceived stress levels (±SEM) in the stress (SG) and control group (CG) over the study period. Note. t = timepoint; t1 = one year before the exam, t2 = three months prior exam, t3 = one week prior exam and t4 = in the middle of the exam period.

2.3. Association between right medial prefrontal cortex (mPFC) response and perceived stress

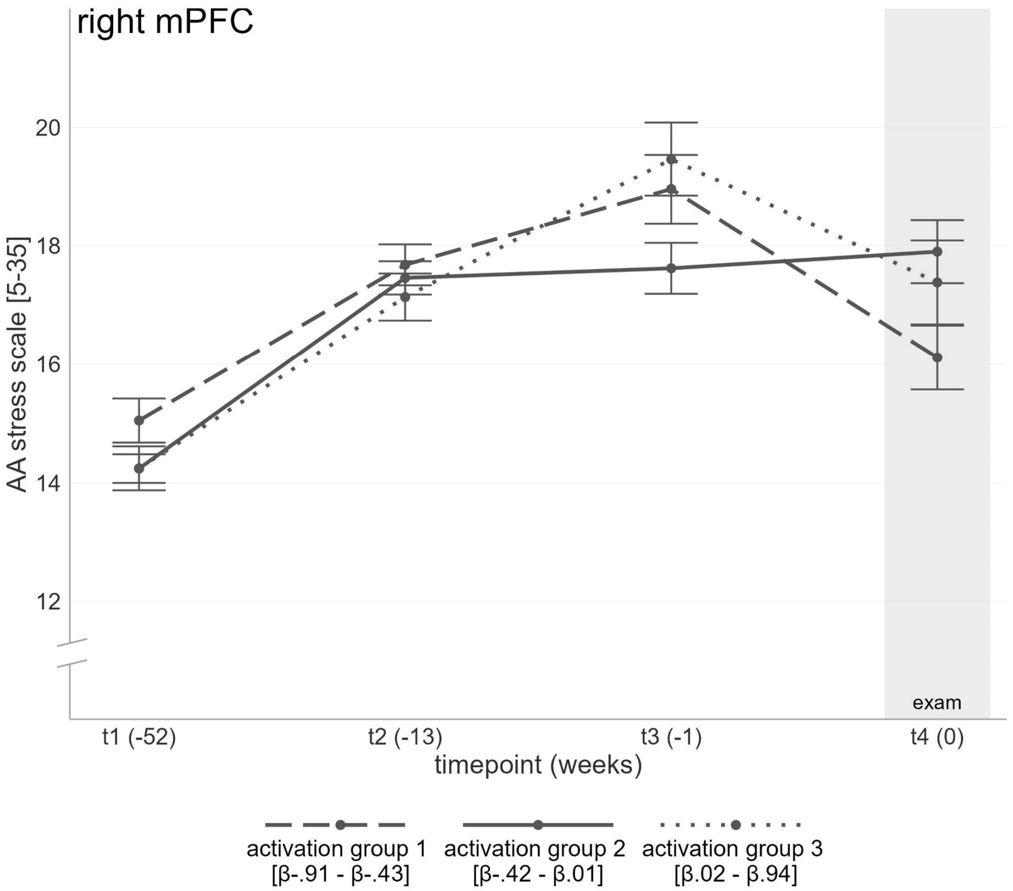

Figure S2. Time course of the *AA stress scale* (±SEM) in the stress group. For illustrative purposes, participants were divided into three groups of equal size according to their right mPFC response. *Note.* t = timepoint; t1 = one year before the exam, t2 = three months prior exam, t3 = one week prior exam and t4 = in the middle of the exam period.

2.4. Cortisol awakening response

Table S3. Parameter estimates for overall effects of the final model for the cortisol awakening response in the stress group.

**Fixed Effects**	**Estimate**	***SE***	***p***
Intercept	0.73	0.06	**< .001**
30 min	0.39	0.05	**< .001**
45 min	0.42	0.06	**< .001**
T2	-0.04	0.04	.337
T3	0.01	0.04	.792
T4	0.03	0.04	.434
T5	0.00	0.04	.944
T6	0.01	0.04	.865
T2 x 30 min	-0.02	0.04	.597
T2 x 45 min	-0.00	0.04	.991
T3 x 30 min	-0.06	0.04	.088
T3 x 45 min	-0.07	0.04	.083
T4 x 30 min	-0.12	0.04	**.002**
T4 x 45 min	-0.18	0.04	**< .001**
T5 x 30 min	-0.01	0.04	.725
T5 x 45 min	-0.03	0.04	.443
T6 x 30 min	-0.02	0.04	.654
T6 x 45 min	-0.02	0.04	.587
**Covariates**			
Women using HC	0.07	0.07	.296
Women using HC x 30 min	-0.15	0.05	**.004**
Women using HC x 45 min	-0.17	0.06	**.005**
Men	0.05	0.07	.475
Men x 30 min	-0.13	0.05	**.015**
Men x 45 min	-0.18	0.06	**.003**
Awakening time	0.10	0.03	**.002**
Awakening time x 30 min	-0.06	0.03	.060
Awakening time x 45 min	-0.08	0.04	**.036**

**Random Effects**	***SD***	Correlation	
		(Intercept)	30 min
Subject (Intercept)	0.12		
30 min	0.08	-.62	
45 min	0.09	-.78	.99
Timepoint (Intercept)	0.18		
30 min	0.15	-.62	
45 min	0.18	-.68	1.00
Residual	0.10		

Note. SE = Standard error; *SD* = Standard deviation; Min = Minutes after awakening; T = Timepoint; HC = Hormonal contraception.

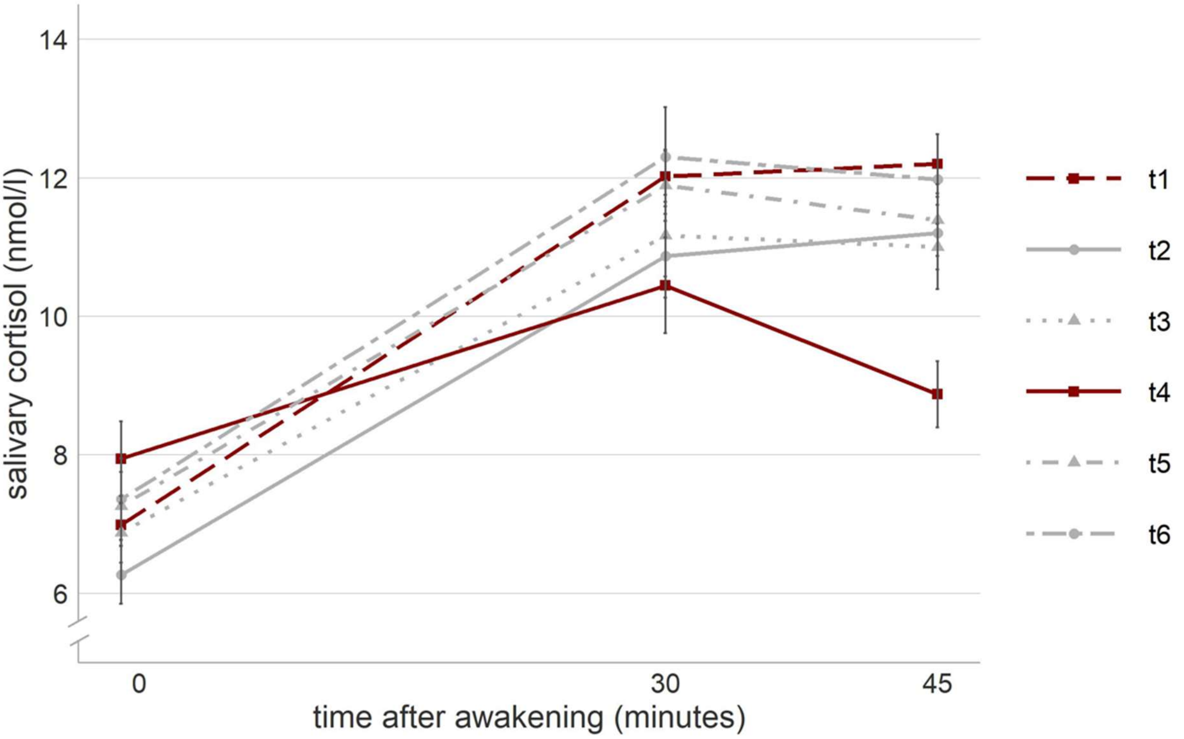

Figure S3. Mean cortisol values (±SEM) for the stress group over the study period. *Note.* t = timepoint; t1 = one year before the exam, t2 = three months prior exam, t3 = one week prior exam, t4 = in the middle of the exam period, t5 = one week after the exam, and t6 = one month after the exam.

References

1. Watson, D., Clark, L. A. & Tellegen, A. Development and validation of brief measures of positive and negative affect: the PANAS scales. *J Pers Soc Psychol.* **54**, 1063 (1988).
2. Steyer, R., Schwenkmezger, P., Notz, P. & Eid, M. *Der Mehrdimensionale Befindlichkeitsfragebogen (MDBF)*. (Hogrefe, 1997).
3. Laux, L., Glanzmann, P., Schaffner, P. & Spielberger, C. D. *Das State-Trait-Angstinventar*. (Beltz, 1981).
4. Powell, D. J. & Schlotz, W. Daily life stress and the cortisol awakening response: testing the anticipation hypothesis. *PloS one* **7**, e52067 (2012).
5. Broderick, J. E., Arnold, D., Kudielka, B. M. & Kirschbaum, C. Salivary cortisol sampling compliance: comparison of patients and healthy volunteers. *Psychoneuroendocrinology* **29**, 636-650 (2004).
6. Kudielka, B. M., Broderick, J. E. & Kirschbaum, C. Compliance with saliva sampling protocols: electronic monitoring reveals invalid cortisol daytime profiles in noncompliant subjects. *Psychosom Med* **65**, 313-319 (2003).
7. Dressendörfer, R., Kirschbaum, C., Rohde, W., Stahl, F. & Strasburger, C. Synthesis of a cortisol-biotin conjugate and evaluation as a tracer in an immunoassay for salivary cortisol measurement. *J Steroid Biochem Mol Biol* **43**, 683-692 (1992).
8. Streit, F. *et al.* A functional variant in the neuropeptide S receptor 1 gene moderates the influence of urban upbringing on stress processing in the amygdala. *Stress* **17**, 352-361 (2014).
9. Henze, G.-I. *et al.* Increasing deactivation of limbic structures over psychosocial stress exposure time. *Biol Psychiatry Cogn Neurosci Neuroimaging* **5**, 697-704 (2020).
